# Which Path to Universal Health Coverage? Perspectives on the World Health Report 2010

**DOI:** 10.1371/journal.pmed.1001001

**Published:** 2010-11-22

**Authors:** Sara Bennett, Sachiko Ozawa, Krishna D. Rao

**Affiliations:** 1 Johns Hopkins Bloomberg School of Public Health, Baltimore, Maryland, United States of America; 2 Public Health Foundation of India, New Delhi, India

From the US to China, South Africa to India, governments and citizens are engaged in an active debate about how best to protect people from catastrophic health care costs while ensuring access to health care of adequate quality. While it is widely known that in the US there are 50 million uninsured, it is perhaps less well known that across the world 100 million people are pushed into poverty each year because of health care expenditures. This is an avoidable tragedy.

With the financial crisis still looming over many countries, this year's World Health Report: “Health Systems Financing: The path to universal coverage” [1] could not be more timely in addressing the question of how to ensure that all people have access to health care services, without suffering financial hardship. In a hundred easy-to-read pages, the report synthesizes evidence concerning effective strategies to achieve universal coverage, drawing upon the now substantial body of published literature from around the world, as well as a number of background papers commissioned especially for the report.

## Steps to Achieving Universal Coverage

The report structures its arguments around three main themes: (i) raising sufficient resources for health, (ii) reducing financial risks and barriers to care, and (iii) increasing efficient use of resources. On the first point, all countries face constant demands to increase their health spending. The report argues that there are multiple ways to do this through leveraging greater domestic resources, notably through exploring an increasing menu of innovative mechanisms to increase funds available to health. However, it also recognizes that most low-income countries need external assistance to achieve universal coverage.

On the basis of the evidence available, the report makes strong policy recommendations to reduce direct payments for health care through risk pooling and prepayment. It notes that countries that have come closest to ensuring universal health coverage mandate contributions for people who can afford to pay, through taxation and/or insurance contributions. It documents the highly negative consequences of user fees (“User fees have punished the poor,” says Margaret Chan, Director General of the World Health Organization in the Preface), and makes a strong push for direct payments to be reduced, whilst carefully considering the accrued evidence and avoiding an absolutist position on this issue. The report argues that there is no single pathway or “magic bullet” for universal coverage. Each country needs to determine for itself an appropriate balance between extending coverage to more people, offering more services, and/or covering more of the cost of care. Further, strategies to achieve these goals also need to be uniquely tailored to reflect country circumstances. The report also seeks to end the rather futile debate about the relative advantages of tax-based versus social health insurance models of paying for health care (sometimes referred to as the Beveridge and Bismark models, respectively). It acknowledges that in reality many countries appropriately use a mix of mechanisms to finance health care.

Finally, in terms of efficiency, the report estimates that 20%–40% of all health spending is currently wasted through inefficiency – a stunning statistic considering how little is spent on health in many countries. Different sources of inefficiency are considered and a range of options for reducing waste is presented. Importantly, the report also identifies the inefficiencies arising from fragmentation in ways that donor funds are delivered to low income countries, and calls for an intensification of efforts to address this problem.

These are important messages that should resonate among all those concerned with improving population health, and guide future policy and decision making (Box 1).

**Box 1. Key Recommendations of the World Health Report 2010**

- There is no single path or magic bullet to achieve universal health coverage: each country needs to devise its own route to achieve this goal.
- All countries, but particularly poorer ones, need to reduce reliance on direct, out-of-pocket payments for health care by increasing risk pooling and prepayment for services.
- Countries should address barriers to health care other than direct payments for care: transport costs and lost income can be substantial obstacles to care seeking.
- There is substantial scope to raise further domestic resources for health care, particularly through innovative approaches to financing.
- 20%–40% of health care expenditure is wasted; improved health system efficiency can make a substantial contribution to the achievement of universal health coverage.
- Wealthier countries should provide financial support to low income countries in order for them to achieve universal health care coverage.
- Despite some progress, development assistance for health remains fragmented and unpredictable; efforts to improve the efficiency and coordination of aid must be intensified.

## Specifying Steps on the Path to Universal Coverage

The report makes many sensible and balanced suggestions and provides an excellent overview of recent evidence. But will it make a difference to the 100 million people potentially facing poverty because of health care costs, or the many other millions without adequate access to care? On this question, our response is nuanced: we believe that the report provides a vision of a good health financing system that is sufficiently flexible for many states to adopt. It also provides a compass and an overview of possible routes to achieve that vision. However, the report falls short in identifying important milestones along the route or providing detailed guidance on the implementation process to achieve universal coverage.

What are appropriate intermediate goals en route to universal coverage? The report discusses the need to extend coverage to the population who are currently not covered, include additional health services, and reduce cost sharing and fees. However, these strategies are unlikely to be of equal importance. In the spirit of the Alma-Ata Declaration, extending population coverage particularly to the underserved, poor, and vulnerable must be the first step along this path. Rather than deepen coverage for those who already have access to health care, we believe it is far more important to direct effort and resources to broaden coverage so that everyone at least has access to affordable medicines and basic health services.

The report leaves open the many different paths that could be taken to broaden coverage. It recognizes the tension between extending coverage to formal sector workers who are easy to reach and better able to pay for health care, versus covering those most in need. It cautions countries against exacerbating societal inequities by providing coverage first to the formal sector. Community health insurance is discussed as a possible institutional stepping stone, but requires strong government leadership to consolidate the schemes. The report also suggests that financing systems are more sustainable where there is a clear link between contribution and benefit. To policy makers trying to decide how best to extend health services to the poor, additional practical advice may be warranted.

The limited discussion on practical implementation issues is perhaps the report's weakest link. In the report's final chapter “An agenda for action,” implementation is dealt with in a rather glib and linear fashion: establish a vision, do a situation analysis, conduct a financial assessment, then develop a strategy for change. The strategies described in the report, however, entail real implementation challenges. For example, the report helpfully describes an array of strategies to address inefficiency in the health sector including: improving the use of medicines, reducing the cost of medicines, rationalizing the use of technology, motivating health workers, improving the efficiency of hospital care, preventing fraud and corruption, and getting the mix of health interventions right. None of these strategies represent startlingly new ideas, so why have we failed to stamp out the problems of waste previously, and how can we be sure that a new wave of efficiency related interventions will be effective?

In many instances, the barriers to universal coverage, or even to just increasing efficiency, are not technical ones but political ones [2],. Even if countries can establish a vision of how they wish to develop their health financing systems, there may not be adequate political momentum to bring this about, or the steps to the achievement of this vision may be blocked by powerful political actors. Some insurance firms may want to see a clear role for private insurers, while some physicians may not want to release their grip on a profitable professional monopoly by task shifting to nurse practitioners or other suitably qualified cadres. Anyone who has lived through the 2010 US health care reform process observed this first hand. Inadequate political support may not prevent reform, but it is likely to prevent the linear step-wise achievement of goals, and frequently lead to second best outcomes. Health financing reform involves trade-offs between different groups. The World Health Report 2010 is sadly silent on the importance of bringing together the political and technical to achieve the grand goal of universal health care (Box 2).

**Box 2. Remaining Questions**

- How can countries direct effort and resources to broaden coverage so that everyone at least has access to affordable medicines and basic health services?
- How best can countries expand risk pooling through mandatory coverage of specific population groups, without focusing on formal sector employees first and thus widening inequities between different societal groups?
- How should countries manage the complex political challenges inherent in health financing reform and marry technical know-how with strategic political thinking?
- What are the underlying obstacles to improving efficiency in country health systems and how can these best be addressed?
- What challenges do the growing burden of chronic diseases and life-style–related illnesses place upon risk pools?
- How can donors be made more accountable to achieve commitments set out in the Paris Declaration on Aid Effectiveness and the Accra Agenda for Action [4]?

## Towards Implementation

There is an explosion of interest in universal health coverage with many countries experimenting with and adopting new health financing strategies. The World Health Organization and other concerned agencies need to ensure that countries can receive support along the path to universal coverage if they seek it. All countries, regardless of their stage of development, face the challenge to increase domestic funds for health care and reduce inefficiencies. Facilitating the sharing of experiences between countries in terms of the challenges faced and how they have been managed is essential. The indicators for monitoring universal coverage presented in the report can help track progress and allow countries to learn which strategies are effective.

The wealth of empirical evidence that the World Health Report 2010 has compiled to inform and support decision making on health financing systems marks a “coming of age” for the field. However, it is clear that much more needs to be done to inform the difficult implementation and political choices that governments face as they tread the path towards universal coverage.
